# Predictive Clinical Neuroscience Portal (PCNportal): instant online access to research-grade normative models for clinical neuroscientists.

**DOI:** 10.12688/wellcomeopenres.19591.1

**Published:** 2023-07-26

**Authors:** Pieter Barkema, Saige Rutherford, Hurng-Chun Lee, Seyed Mostafa Kia, Hannah Savage, Christian Beckmann, Andre Marquand

**Affiliations:** 1 Radboud University, Donders Institute for Brain, Cognition and Behaviour, Nijmegen, The Netherlands; 2 Department of Cognitive Neuroscience, Radboud University Medical Centre, Nijmegen, The Netherlands; 3 Department of Psychiatry, University of Michigan, Ann Arbor, USA; 4 Department of Psychiatry, Utrecht University Medical Center, Utrecht, The Netherlands; 5 Department of Cognitive Science and Artificial Intelligence, Tilburg University, Tilburg, The Netherlands; 6 Centre for functional MRI of the Brain, University of Oxford, Oxford, England, UK

**Keywords:** braincharts, normative modelling, brain growth charting, PCNtoolkit

## Abstract

**Background: **The neurobiology of mental disorders remains poorly understood despite substantial scientific efforts, due to large clinical heterogeneity and to a lack of tools suitable to map individual variability. Normative modeling is one recently successful framework that can address these problems by comparing individuals to a reference population. The methodological underpinnings of normative modelling are, however, relatively complex and computationally expensive. Our research group has developed the python-based normative modelling package Predictive Clinical Neuroscience toolkit (PCNtoolkit) which provides access to many validated algorithms for normative modelling. PCNtoolkit has since proven to be a strong foundation for large scale normative modelling, but still requires significant computation power, time and technical expertise to develop.

**Methods: **To address these problems, we introduce PCNportal. PCNportal is an online platform integrated with PCNtoolkit that offers access to pre-trained research-grade normative models estimated on tens of thousands of participants, without the need for computation power or programming abilities. PCNportal is an easy-to-use web interface that is highly scalable to large user bases as necessary. Finally, we demonstrate how the resulting normalized deviation scores can be used in a clinical application through a schizophrenia classification task applied to cortical thickness and volumetric data from the longitudinal Northwestern University Schizophrenia Data and Software Tool (NUSDAST) dataset.

**Results: **At each longitudinal timepoint, the transferred normative models achieved a mean[std. dev.] explained variance of 9.4[8.8]%, 9.2[9.2]%, 5.6[7.4]% respectively in the control group and 4.7[5.5]%, 6.0[6.2]%, 4.2[6.9]% in the schizophrenia group. Diagnostic classifiers achieved AUC of 0.78, 0.76 and 0.71 respectively.

**Conclusions: ** This replicates the utility of normative models for diagnostic classification of schizophrenia and showcases the use of PCNportal for clinical neuroimaging. By facilitating and speeding up research with high-quality normative models, this work contributes to research in inter-individual variability, clinical heterogeneity and precision medicine.

## Introduction

The neurobiological basis of mental disorders remains poorly understood despite large scientific efforts. A major factor in this knowledge gap has been the strong focus on group average effects which largely neglect individual differences in brain structure or function. As a consequence, most analytical approaches classically used to study mental disorders assume that subjects with the same diagnostic label neurobiologically deviate in the same way; a misleading assumption.

Normative modelling is one recently successful framework that can address this problem ( Marquand *et al*., 2019). Normative modelling detects individual-level differences by placing each person into the reference population, producing individualized deviation scores. Applied to brain data, the magnitude and spatial pattern of individual brain deviations can then be linked to the severity of symptoms, psychiatric diagnosis, or other behavioral characteristics. Researchers from various clinical and non-clinical fields could benefit from adopting this framework to facilitate valid inference on the individual brain level, paving the way towards precision medicine.

The methodological underpinnings of normative modelling are, however, relatively complex and the estimation of normative models requires access to large datasets processed using consistent pipelines. These factors reduce the accessibility of normative modelling to many seeking its benefits. Our research group has developed the python-based normative modelling package PCNtoolkit ( Marquand *et al.*, 2021) which provides access to many validated algorithms for normative modelling and solutions for accommodating data collection site effects ( Kia *et al*., 2020), non-Gaussian data distributions ( de Boer *et al.*, 2022; Dinga *et al*., 2021; Fraza *et al*., 2021), federated (i.e. decentralized) model estimation ( Kia *et al*., 2022) and other statistical problems in a consistent and principled way.

While normative modelling has been mainly a tool for data scientists, the increase in appreciation for individual brain differences in clinical research comes with an increase in demand from users without a background in computer science and programming. Recent applications illustrate the growing success of normative modelling, such as in Alzheimer’s Disease ( Verdi *et al*., 2023) and schizophrenia ( Lv *et al*., 2021; Wolfers *et al.*, 2018) and software packages provide the tools necessary to support the increase in demand from a diverse audience. Our software package, PCNtoolkit ( Marquand *et al.*, 2021), for example, has been used to research individuals with autism spectrum disorder, where normative models revealed highly individualized brain development trajectories that cannot be captured by classical ‘case-control’ (group-average) studies ( Zabihi *et al*., 2019). PCNtoolkit has also been used for stratifying attention-deficit/hyperactivity disorder (ADHD) ( Wolfers *et al*., 2020), schizophrenia and bipolar disorder ( Wolfers *et al*., 2021), and general psychopathology ( Parkes *et al.*, 2021). PCNtoolkit has thus proven to be a strong foundation for large scale normative modelling, but – as noted above – still requires significant computation power, time and technical expertise to develop optimized normative models (despite efforts to make the PCNtoolkit more accessible ( Rutherford *et al*., 2022)). There are other tools available that offer code-free normative modelling ( Bethlehem *et al*., 2022; Ge *et al*., 2023), but these tools currently offer a relatively limited range of phenotypes, limited flexibility in terms of estimation methods and are not readily extendible in terms of hosting contributions from other research groups. These constraints leave much potential of normative modelling unfulfilled for the neuroscience community, as demonstrated by our recently published work ( Rutherford *et al*., 2023).

To address these problems, we introduce PCNportal. PCNportal is an online platform integrated with PCNtoolkit that: (i) offers access to pre-trained research-grade normative models estimated on tens of thousands of participants, without the need for computation power or programming abilities – only a dataset and an internet connection and (ii) provides a platform that enables researchers to easily share pre-estimated normative models with the community. We currently support models for estimates of regional brain volume, cortical thickness, surface area and resting state functional connectivity, but our hope is that PCNportal will provide a library of models that are useful in a wide range of neuroscience applications. In more detail, PCNportal is a lightweight client-server application. The client side is an online application ( https://pcnportal.dccn.nl/) with a simple user-friendly graphical user interface. It allows users from all backgrounds, technical, clinical or otherwise, to use high-quality pre-trained models for their research. Our application can easily scale to large amounts of data and users, and aims to be as transparent and responsible as possible with regard to safeguarding data privacy. We believe this increased accessibility of normative models can help to accelerate research into individual variability, not only generally contributing to a better understanding of interpersonal differences in biological features (brain or otherwise), but also benefiting precision medicine for mental illness.

## Methods

**Figure 1.**
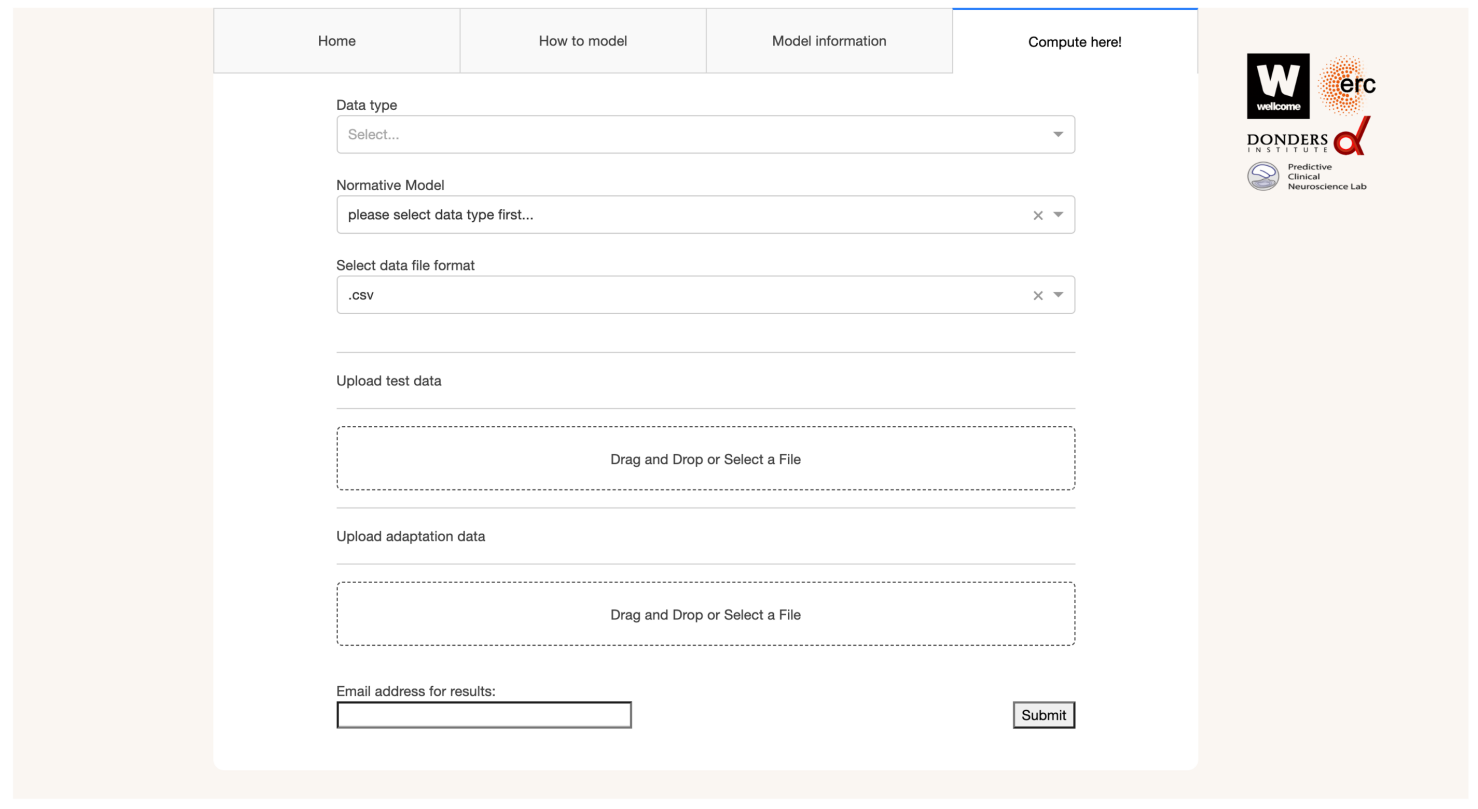
The intuitive web-based graphical user interface of PCNportal allows users to easily adjust and apply validated pre-trained normative models to model their data on the individual level. Other website tabs provide demos and guidelines to help the user.

PCNportal is built as an extension of PCNtoolkit that allows users to easily apply normative models pre-trained on large neuroimaging (i.e. ‘reference’) datasets to a brain imaging dataset of choice without needing programming code or computing power. The reference datasets cited here were derived by aggregating large numbers of publicly available data, e.g. N=58,836 from 82 scanning sites ( Rutherford *et al*., 2022), N=43,524 from 66 sites ( Rutherford *et al*., 2023) or 37,128 from 79 sites ( Kia *et al*., 2022) . In more detail, PCNportal is a lightweight client-server application. The client side is an online application ( https://pcnportal.dccn.nl/) with a simple user-friendly graphical user interface (shown in Figure 1). It contains all instructions and information necessary to quickly get started with modeling, but also refers to elaborate tutorials (e.g. https://pcntoolkit.readthedocs.io/en/latest/) and published work using these models to promote a deeper understanding of the subject matter (e.g.( Fraza *et al.*, 2021; Kia *et al.*, 2020)). On the server side, a set of helper scripts integrate the online application with a back-end, based on the PCNtoolkit library, which performs the computations necessary to adapt the pre-trained model to the new dataset (via transfer learning) and distribute the computational workload across our dedicated computation nodes to reduce modelling time. PCNportal then uploads the (anonymized) results to a publicly accessible server hosted on an academic platform (SURFdrive) and shares them with the user through email. Lastly, it automatically deletes all user data after a thirty-day period. Before that time, data is only accessible by a small group of core developers.

### Implementation

**Figure 2.**
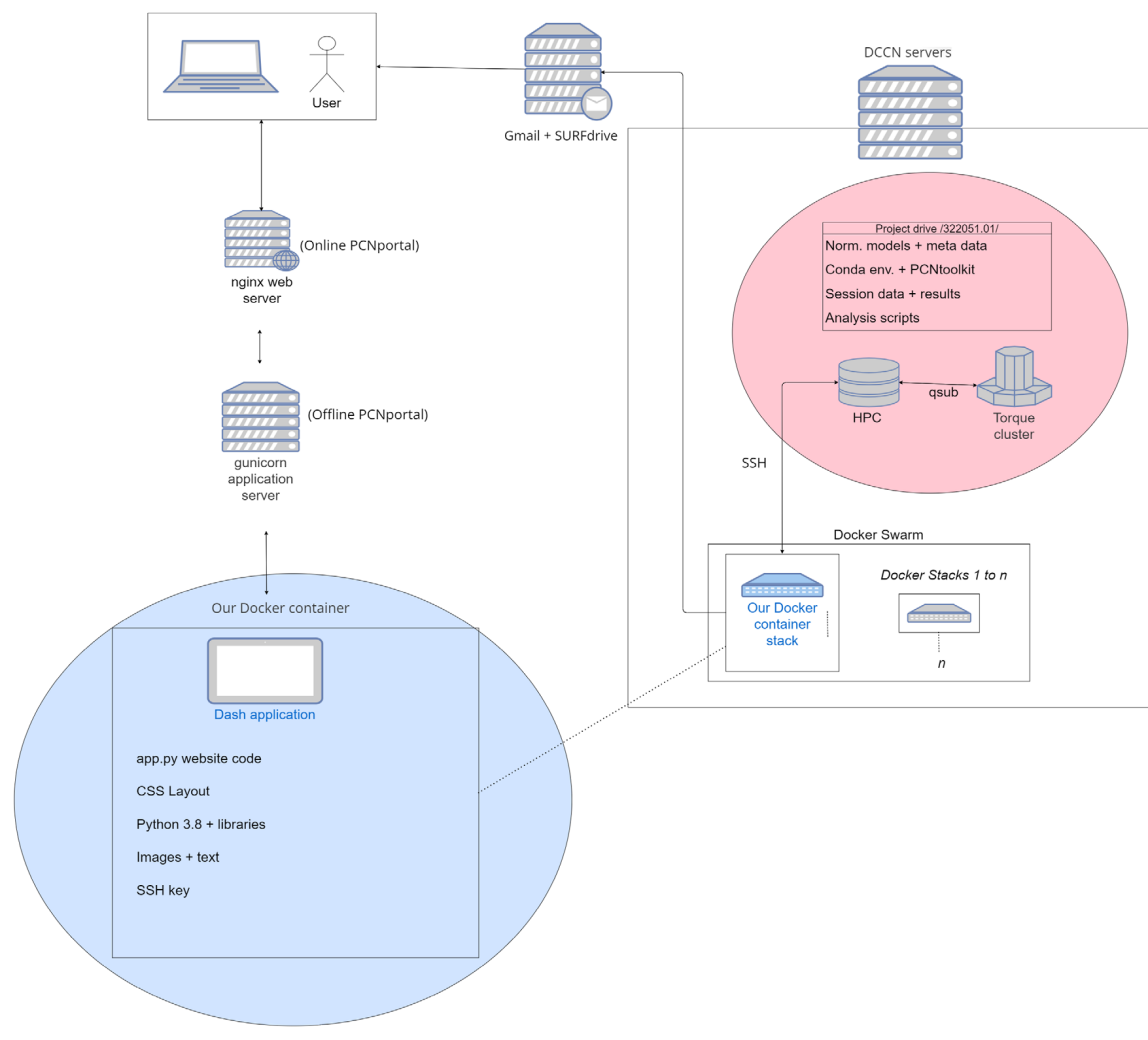
A network diagram showing the technical infrastructure used to support PCNportal. The client-side web interface is hosted from a Docker container that lives in the servers of the Donders Center for Cognitive Neuroimaging, where it can efficiently model data with parallelized cluster computers.

The design of PCNportal specifically keeps in mind the growing demand for normative models by being highly scalable and flexible. From a technical perspective, our web platform is a Flask-based application hosted through the gunicorn server ( https://docs.gunicorn.org/en/stable/) and nginx proxy ( https://docs.nginx.com/) that support scaling up to large user bases. PCNportal is currently hosted within the technical infrastructure of the Donders Center for Cognitive Neuroimaging, where clusters dedicated to mass computation can simultaneously process many user requests. The application is containerized with Docker to make sure that our application can be maintained with ease, has minimum downtime and is highly portable, ensuring that it can be scaled up to cloud-based platforms as necessary. Results are hosted through SURFdrive, a private and university-regulated sharing platform, and sent through Google’s gmail, where any personal information is immediately deleted. PCNportal runs automated scripts in the background to remove results older than thirty days to aim for compliance with privacy guidelines such as the General Data Protection Regulation, however it should be noted that users of PCNportal retain the responsibility to ensure that they have permission to share (pseudo-) anonymized datasets that are uploaded to the system. The architecture of PCNportal is shown in Figure 2, below.

### Operation

***Workflow.*** The only requirement for using PCNportal is an up-to-date web browser such as Chrome or Firefox, although older versions may still work. The workflow consists of data formatting, submitting and downloading results. First, the user chooses the data type they want to model. Then, they can explore the various modelling options by clicking on them and exploring model-related information and hyperparameters. Having chosen the model, the user can download a template that describes the inputs to the model (e.g. a comma-separated variable file with specific column names). The user will need to format their data according to this data template and can submit the data along with their model of choice and email address. The back-end server then adapts the model parameters to match the user’s data using a transfer learning paradigm (e.g. in order to accommodate site effects for sites that are not present in the training data). Importantly, this adaptation is done using a held-out partition of the data in order to avoid biasing downstream analysis. Out-of-sample performance metrics and subject-level deviation scores are computed, and finally, a mail will be sent to the user to notify them that the results of their model adaptation are ready to download. This can take anywhere from approximately half an hour to a day, depending on the model and data set size.

***Model contributions.*** Currently, PCNportal provides access to existing normative models based on cortical thickness, brain volume, surface area and resting-state functional connectivity ( Kia *et al*., 2020; Kia *et al*., 2022; Rutherford *et al.*, 2022; Rutherford *et al.*, 2023), but we have designed this application to facilitate straightforward contribution of additional models. Flexibly increasing the number of available models is a crucial design feature, as normative models can be developed for many different data types, other than brain images, including but not limited to genetic data, psychophysiological data and digital phenotyping data, with applications to neuropsychiatry and beyond. We therefore designed PCNportal in a way that new models can be dynamically added and are instantly available to users. We also welcome model contributions from other groups that can be added to the website after validation tests. For example, we plan to host the fine-grained normative models for the cerebellum reported in ( Gaiser *et al*., 2023) and voxel-wise models of functional MRI data ( Savage *et al*., 2023) sin the coming months.

## Use cases

PCNportal can be used to compare a user’s data set to a reference normative model with minimum effort, accounting for site effects, and covariates such as age and sex. The resulting normalized data can then, for example, serve clinical applications. In this example, we will demonstrate how PCNportal can be used to model a demo data set into deviation scores and use them in a clinical classification task for schizophrenia. A tutorial of the exact steps to model data in PCNportal can be found on the bottom of the ‘How to model’ tab on https://pcnportal.dccn.nl/. We have extensively validated and demonstrated the types of analyses that can be conducted with PCNportal in prior work ( Rutherford *et al.*, 2023), but here we choose a simple illustrative application, whereby we replicate one of these findings in this paper, using an independent dataset.

### Model

For this example, we choose a normative model using the Bayesian Linear Regression algorithm, pre-trained on the average cortical thickness of brain regions from 58,836 samples from 82 data collection sites, previously published in Rutherford, Fraza *et al*., 2022. For full details on the validation and benchmarking of this model, please refer to Rutherford *et al.* (2023). However, briefly, the age range of this sample was 2-100 with an approximate balance of males and females. This dataset was derived by aggregating mostly publicly available datasets, including UKBiobank ( Miller *et al*., 2016), the ABCD study ( Volkow *et al*., 2018), the Human Connectome Project lifespan dataset ( Van Essen *et al*., 2013), CamCan, the CMI Healthy Brain Network dataset, the AOMIC dataset ( Snoek *et al*., 2021), Philadelphia Neurodevelopmental Cohort ( Calkins *et al.*, 2015) and the OASIS-3 dataset ( LaMontagne *et al*., 2019) in addition to a number of smaller public and in-house samples (see ‘data availability' below). Structural MRI data were available for all samples, and all data were processed using the Freesurfer software packgage version 6.0 using the recon-all pipeline with default options. All data included in this sample passed automated quality control checks and a subset of these data were also manually quality checked by experienced raters. Finally, parcellated cortical thickness data from the ‘Destrieux’ atlas and subcortical volumes from the Freesurfer subcortical pipeline. A normative model was fit to data from each cortical or subcortical parcel using the PCNtoolkit software, using a warped Bayesian linear regression model as described in Fraza *et al*., 2021.

### Data preparation

**Figure 3.**
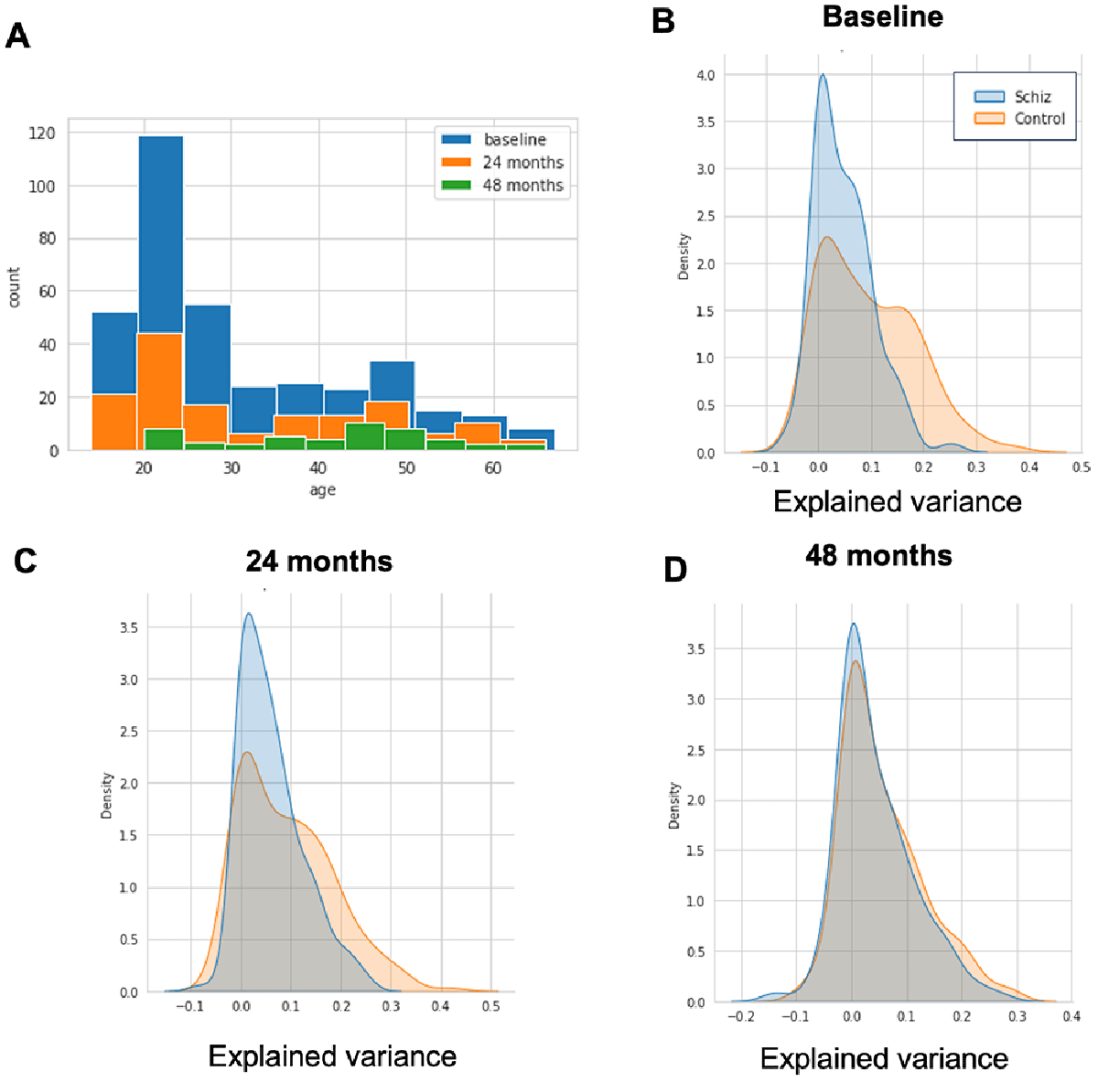
Panel **A**: The age distribution for individuals Northwestern University Schizophrenia Data and Software Tool (NUSDAST) dataset used to demonstrate the usage of PCNportal. Panels **C**– **D**: Proportion of explained variance for the normative models transferred to the NUSDAST sample for the baseline visit ( **A**), 24-month follow-up ( **B**) and 48-month follow up ( **C**).

To prepare the input, we first need to prepare our data to match the model template, by containing the same image-derived phenotype (IDP) names and the necessary covariates (for our model: ‘sex’, ‘site’ and ‘age’). This is, in practice, specified as a csv. We have chosen a subset of the publicly available Northwestern University Schizophrenia Data and Software Tool (NUSDAST) data set ( Wang *et al.*, 2013), containing data high resolution magnetic resonance imaging (MRI) datasets from individuals with schizophrenia, their non-psychotic siblings, in addition to healthy controls, all on the same MRI scanning platform. Please see Wang *et al*., 2013 for details surrounding the recruitment, diagnosis and MRI scanning procedures and also see our ‘Data Availability’ statement for where to download this openly accessible data. The NUSDAST data set contains longitudinal neuroimaging measurements across three time points: at the start of the study and after 24 and 48 months although for simplicity we process each timepoint independently in this manuscript. The age distribution of this sample is summarized in Figure 3A for each longitudinal wave. We processed all datasets using Freesurfer version 6.0 in order to derive estimates of cortical thickness and subcortical volumes, using identical procedures to what we have reported previously ( Rutherford *et al.*, 2022) pipelines. We created an adaptation set of the same forty controls from the baseline timepoint to adjust for the scanner effect and test the adapted normative model on the remaining data per scanning moment. We can then run PCNportal to obtain normalized deviation scores, with respect to the normative reference model provided in Rutherford *et al*., 2022. Notably, this step does not require writing any code. We show the explained variance of the transferred models in Figure 3B, C and D.

### Schizophrenia classification with individualized deviation scores

To show how PCNportal could have clinically relevant applications, we demonstrate a use case in which the resulting deviation scores are used for a classification task between schizophrenia patients and controls. For this analysis, we classify subjects at three time points: at the start of the study and after respectively 24 and 48 months, thereby aiming to replicate the accuracy obtained in ( Rutherford *et al*., 2023). We train a Support Vector Machine on the data using ten-fold cross-validation and a linear kernel with default parameters, following this work.

**Figure 4.**
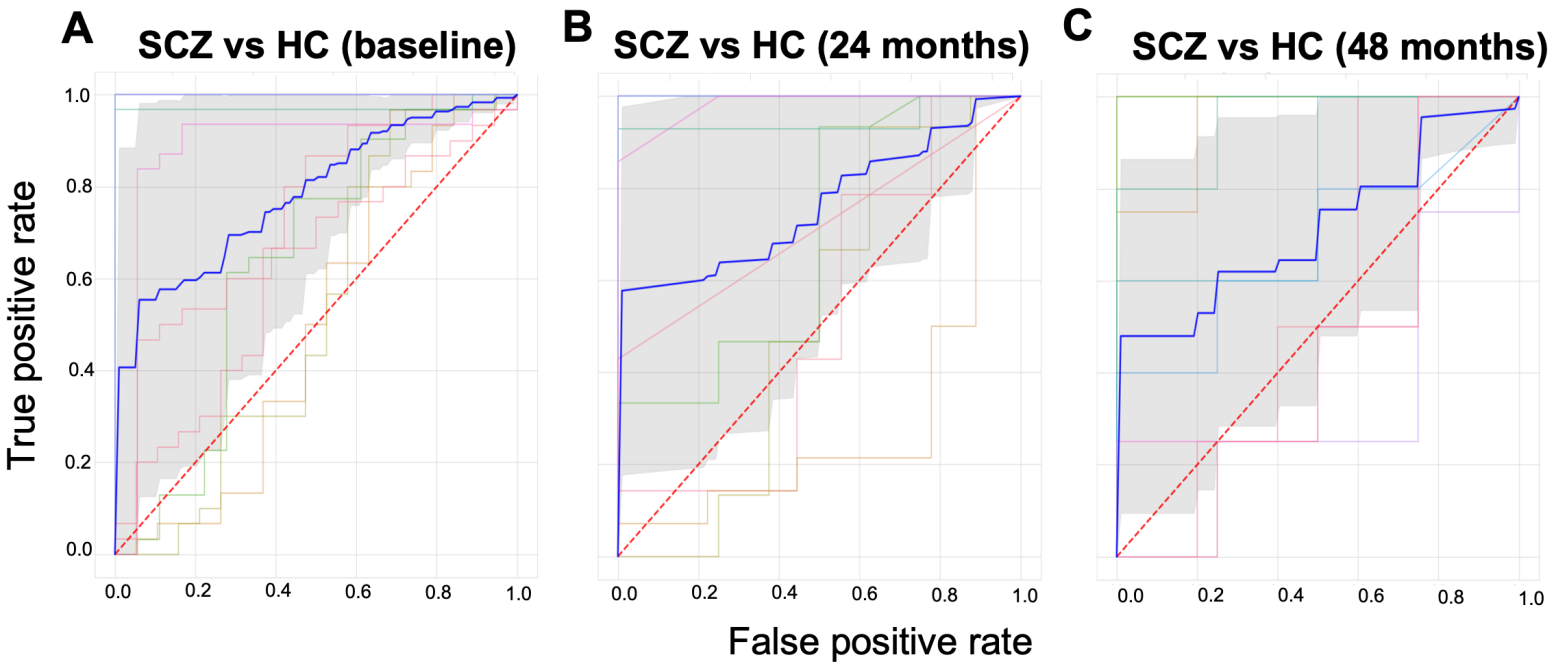
We trained a Support Vector Machine to classify subjects of the NUSDAST data set into individuals with schizophrenia or controls on three sequential time points. The data at the first time point consisted of 183 patients and 306 controls. The model obtained an area under the receiver operating characteristic curve (AUROC) = 0.78. At time point 2, after 48 months with 82 patients, 142 controls, the model obtained AUROC = 0.76. And at time point 3 (48 months), with 43 patients and 44 controls, an AUROC = 0.71. The bold blue line shows the mean ROC curve and the lighter-coloured lines show the ROC curves for each of the cross-validation folds.

This simple analysis illustrates the utility of PCNportal and shows diagnostic accuracies in the range as we have reported previously across all imaging timepoints ( Figure 4). We nevertheless emphasize that this analysis is only illustrative, not an approved tool for clinical applications.

## Conclusion

Through PCNportal, research efforts towards conceptualizing mental illness can directly benefit from fine-tuned and well-validated models developed by research labs specialized in normative modelling, without requiring specialized technical expertise or infrastructure. We hope that the open-source nature of PCNportal will hopefully serve as an inspiration to other scientific teams that wish to share their big data models and technological infrastructure with a wider audience. Altogether, PCNportal provides freely accessible high-quality brain data analysis on the individual level for a wide range of neural biomarkers to the research community in a scalable way, with ongoing projects to support the modeling of data types beyond brain images. By bridging the gap between methodological development and urgent clinical challenges, we believe our contribution builds toward a better understanding of neurological or biological differences between individuals that in turn supports the understanding and treatment of mental and physical disorders.

## Data Availability

The data availability for training the online normative models is described in the associated publications. Kia *et al*., 2022 (publicly available and in-house neuroimaging data from ABCD, ABIDE, CamCan, CMI, CNP, FCON, HCPAG, HCPDV, IXI, NKI, AOSIS, NKI, OASIS, OPN, PNC, UKB and TOP consortia) https://doi.org/10.1371/journal.pone.0278776 Rutherford *et al*., 2022 (publicly available and in-house neuroimaging data from ABCD, ABIDE, AOMIC CamCan, CMI, CNP, Delta, FCON, HCPAG, HCPDV, IXI, NKI, AOSIS, NKI, OASIS, OPN, PNC, UKB, UMICH MTwins, UMICH CWS, UMICH SAD, UMICH SZGand TOP consortia) https://doi.org/10.1101/2022.10.05.510988 Rutherford *et al.*, 2023 (publicly available and in-house neuroimaging data from ABCD, ABIDE, AOMIC CamCan, CMI, CNP, Delta, FCON, HCPAG, HCPDV, IXI, NKI, AOSIS, NKI, OASIS, OPN, PNC, UKB, UMICH MTwins, UMICH CWS, UMICH SAD, UMICH SZG and TOP consortia) https://doi.org/10.7554/eLife.85082 The ABCD data are available at https://nda.nih.gov/abcd/ The ABIDE data are available at https://fcon_1000.projects.nitrc.org/indi/abide/ The AOMIC data are available at https://nilab-uva.github.io/AOMIC.github.io/ The CamCAN data are available at https://www.cam-can.com/ The CMI data are available at at https://www.oasis-brains.org/ http://fcon_1000.projects.nitrc.org/indi/cmi_healthy_brain_network/sharing_neuro.html. The CNP data are available at https://legacy.openfmri.org/dataset/ds000030/ (accession number ds000030). The Delta data are available by reasonable request by contacting Dr. Eric Ruhé at Radboud University Medical Centre ( eric.ruhe@radboudumc.nl) The FCON data are available at http://fcon_1000.projects.nitrc.org/fcpClassic/FcpTable.html. The HCP data are available at https://www.humanconnectome.org/study/hcp-young-adult/document/1200-subjects-data-release. The HCPAG data are available at https://www.humanconnectome.org/study/hcp-lifespan-aging/data-releases. HCPDV data are available at https://www.humanconnectome.org/study/hcp-lifespan-development/data-releases. HCPEP data are available at https://www.humanconnectome.org/study/human-connectome-project-for-early-psychosis. IXI data are available at https://brain-development.org/ixi-dataset/. NKI data are available at https://fcon_1000.projects.nitrc.org/indi/pro/nki.html. OASIS3 dataset is available at https://www.oasis-brains.org/. The OASIS dataset is available at The OPN dataset is available at https://openneuro.org/. PNC data are available at https://www.med.upenn.edu/bbl/philadelphianeurodevelopmentalcohort.html. UKBB data are accessible at https://biobank.ctsu.ox.ac.uk/crystal/exinfo.cgi?src=accessing_data_guide. UMICH MTwins data are available by reasonable request at https://sites.lsa.umich.edu/mindlab/families-and-participants/michigan-twin-study-information/ UMICH CWS data are available by request to Dr. Soo Eun Chang sooeunc@umich.edu. UMICH SAD data are available by request to Dr. Elizabeth Duval eduval@umich.edu. UMICH SZG data are available by request to Dr. Ivo Tso tso.23@osu.edu. TOP data are subject to clinical data privacy and are available from the Norwegian Centre for Mental Disorders Research, Institute of Clinical Medicine Institutional for researchers who meet the criteria for access to confidential data. The non-author contact information for the Norwegian Centre for Mental Disorders Research (to which data requests may be sent) is Christine Lycke Brandt( https://www.med.uio.no/klinmed/english/people/aca/chrislyc/index.html), Administrative Manager - NORMENT part UiO, Email: c.l.brandt@medisin.uio.no, Mobile phone: 97118354, Visiting address: Oslo Universitetssykehus HF – Ullevål, Avdeling for psykoseforskning, Bygg 49, 0424 Oslo, Postal address: OUS, Postboks 4956 Nydalen, 0424 Oslo.

## Software availability

- All software used in PCNportal can be found in our public GitHub repository. PCNportal is licensed under the GNU General Public License v3.0.
- Repository: PCNportal. https://doi.org/10.5281/zenodo.7708367 and GitHub: https://github.com/predictive-clinical-neuroscience/PCNportal
- PCNtoolkit is licensed under the GNU General Public License v3.0.
- Repository: https://zenodo.org/record/5207839 and GitHub https://github.com/amarquand/PCNtoolkit
- The software for the classification task was adapted from https://github.com/saigerutherford/evidence_embracing_nm/blob/main/svm_groupdiff_ct_yeo.ipynb.

### Extended data

The Northwestern University Schizophrenia Data and Software Tool (NUSDAST) data set ( Wang *et al*., 2013) is publicly available at http://niacal.northwestern.edu/projects/9.
