# Supplementary material for: Predictive Clinical Neuroscience Portal (PCNportal): instant online access to research-grade normative models for clinical neuroscientists
Source: Wellcome Open Res. 2023 Nov 20;8:326. Originally published 2023 Jul 26. [Version 2] doi: 10.12688/wellcomeopenres.19591.2 (PMC10474337; doi:10.12688/wellcomeopenres.19591.2)
Supplement: Peer review history

# Reviewer report: Reviewer response for version 2

**DOI:** 10.21956/wellcomeopenres.22737.r70285

**Published:** 2023-11-21

**Authors:** Indranath Chatterjee

**Affiliations:** 1 Tongmyong University, Busan, South Korea

Approved

Are the conclusions about the tool and its performance adequately supported by the findings presented in the article?

Yes

Is the rationale for developing the new software tool clearly explained?

Yes

Is the description of the software tool technically sound?

Yes

Are sufficient details of the code, methods and analysis (if applicable) provided to allow replication of the software development and its use by others?

Partly

Is sufficient information provided to allow interpretation of the expected output datasets and any results generated using the tool?

Partly

Reviewer Expertise:

Neuroimaging, Schizophrenia, Computational Neuroscience, Medical Imaging, Deep Learning and AI.

I confirm that I have read this submission and believe that I have an appropriate level of expertise to confirm that it is of an acceptable scientific standard.

# Reviewer report: Reviewer response for version 1

**DOI:** 10.21956/wellcomeopenres.21701.r64530

**Published:** 2023-09-01

**Authors:** Indranath Chatterjee

**Affiliations:** 1 Tongmyong University, Busan, South Korea

The paper is a good contribution to the field. The authors address the challenging issue of understanding the neurobiology of mental disorders by introducing a pioneering tool, PCNportal, that grants immediate access to well-established normative models.

 The introduction effectively outlines the existing limitations in the field due to clinical heterogeneity and the absence of suitable tools for individual variability mapping. The significance of normative modeling is eloquently presented as a solution to these challenges. The authors' work on developing the Predictive Clinical Neuroscience toolkit (PCNtoolkit) showcases their expertise and forms a strong foundation for this study.

 The introduction of PCNportal is a commendable step towards democratizing normative modeling. The platform's integration with PCNtoolkit and its ability to provide access to pre-trained models without the need for computational power or programming skills are particularly noteworthy. The application of PCNportal to a schizophrenia classification task adds practicality and depth to the study, demonstrating its real-world applicability.

 The presented results demonstrate the value of PCNportal's transferred normative models for diagnostic classification. The achieved explained variances and AUC values indicate the reliability of the approach, reinforcing its utility.

**Basic Technical Change:** While the paper is well-structured and insightful, one minor enhancement could be made. Consider briefly elaborating on how PCNportal addresses potential privacy concerns when handling participant data. Assuring users that their data will be handled with utmost privacy and adhering to ethical guidelines could further bolster user confidence in the platform.

 Overall, this paper offers a significant advancement in the field of clinical neuroimaging by simplifying access to research-grade normative models. The authors' comprehensive work deserves appreciation for its potential impact on understanding inter-individual variability, clinical heterogeneity, and precision medicine.

Are the conclusions about the tool and its performance adequately supported by the findings presented in the article?

Yes

Is the rationale for developing the new software tool clearly explained?

Yes

Is the description of the software tool technically sound?

Yes

Are sufficient details of the code, methods and analysis (if applicable) provided to allow replication of the software development and its use by others?

Partly

Is sufficient information provided to allow interpretation of the expected output datasets and any results generated using the tool?

Partly

Reviewer Expertise:

Neuroimaging, Schizophrenia, Computational Neuroscience, Medical Imaging, Deep Learning and AI.

I confirm that I have read this submission and believe that I have an appropriate level of expertise to confirm that it is of an acceptable scientific standard.

# Response

**Published:** 2023-11-17

**Authors:** Andre Marquand

**Affiliations:** Donders Institute for Brain, Cognition and Behavio, Radboud Universiteit, Nijmegen, Gelderland, The Netherlands

***We would like to sincerely thank both reviewers for their careful review of our manuscript and extensive evaluation of the positive and to-be-improved parts of our manuscript and software. We especially thank them for their user-focused feedback which will improve the modelling experience for the target audience. We have now deployed a new release of the software that addresses the reviewers’ comments and we provide a point-by-point response to comments of the reviewer below (our responses in bold text)***  

 Basic Technical Change: While the paper is well-structured and insightful, one minor enhancement could be made. Consider briefly elaborating on how PCNportal addresses potential privacy concerns when handling participant data. Assuring users that their data will be handled with utmost privacy and adhering to ethical guidelines could further bolster user confidence in the platform. **Thank you for this important point. Taken together with the feedback from reviewer 1 and with how seriously we take privacy, we have now devoted a new tab on the website solemnly to data privacy. Here, we aimed to boost user confidence as much as possible by striving to be open and complete about the way we aim to ethically handle user data.**

 Overall, this paper offers a significant advancement in the field of clinical neuroimaging by simplifying access to research-grade normative models. The authors' comprehensive work deserves appreciation for its potential impact on understanding inter-individual variability, clinical heterogeneity, and precision medicine. **Again, we thank the reviewer very much for their appreciation of this work.**

# Reviewer report: Reviewer response for version 1

**DOI:** 10.21956/wellcomeopenres.21701.r64008

**Published:** 2023-08-24

**Authors:** Maria Di Biase

**Affiliations:** 1 Melbourne Neuropsychiatry Centre, Department of Psychiatry, Melbourne Health, The University of Melbourne, Carlton, Victoria, Australia

This manuscript introduces the Predictive Clinical Neuroscience Portal (PCNportal), a valuable online tool providing access to established normative models within a user-friendly, code-free environment. PCNportal effectively streamlines normative model research applications, and the inherent simplicity of the tool guarantees accessibility to diverse neuroscience communities. The technical backbone, including algorithms and data aggregation, were established in prior work. Minor comments here thus focus on enhancing engagement with the manuscript and associated tool.

 The manuscript thoughtfully addresses the critical consideration of data sharing, describing a data retention policy of 30 days. To further instill confidence in users' ability to utilize the tool while adhering to data sharing agreements, it might be beneficial to provide any further explicit assurances, e.g., guarantees that the limited access to data by core developers will strictly uphold privacy and not be utilized for any purpose beyond trouble shooting in cases where users request support.

 The Figure 1 legend could briefly outline the purpose of the "test data" and "adaption data" fields to enhance user understanding and encourage immediate use. More detail regarding these fields and how to select appropriate data could be offered in the PCNportal’s "How to model" tab.

 The PCNportal “compute” tab could incorporate an optional interactive questionnaire to help guide algorithm selection based on the user's data and specific requirements. This could further streamline procedures and help clinical users justify the selection of a specific algorithm in their publications.

 The brain atlas used for normative modeling and associated template csv files could be stated in the “How to model tab” to help users organise their regional surface outputs as suitable PCNportal inputs. 

 To further cater to the clinical user base, it is suggested that the outputs generated by the PCNportal be presented in csv instead of pickle files to facilitate seamless integration of the generated insights into other—non-python-based—workflows. Furthermore, each of the 7 outputs could be briefly explained in the “How to model” tab. 

 To ensure proper acknowledgement, the “compute” tab could include specific citations that should be referenced in reports utilizing this tool.

Are the conclusions about the tool and its performance adequately supported by the findings presented in the article?

Yes

Is the rationale for developing the new software tool clearly explained?

Yes

Is the description of the software tool technically sound?

Yes

Are sufficient details of the code, methods and analysis (if applicable) provided to allow replication of the software development and its use by others?

Yes

Is sufficient information provided to allow interpretation of the expected output datasets and any results generated using the tool?

Partly

Reviewer Expertise:

Neuroimaging, stem cell disease modeling, neuroscience & psychiatry

I confirm that I have read this submission and believe that I have an appropriate level of expertise to confirm that it is of an acceptable scientific standard.

# Response

**Published:** 2023-11-17

**Authors:** Andre Marquand

**Affiliations:** Donders Institute for Brain, Cognition and Behavio, Radboud Universiteit, Nijmegen, Gelderland, The Netherlands

***We would like to sincerely thank both reviewers for their careful review of our manuscript and extensive evaluation of the positive and to-be-improved parts of our manuscript and software. We especially thank them for their user-focused feedback which will improve the modelling experience for the target audience. We have now deployed a new release of the software that addresses the reviewers’ comments and we provide a point-by-point response to comments of both reviewers below.***

 The manuscript thoughtfully addresses the critical consideration of data sharing, describing a data retention policy of 30 days. To further instill confidence in users' ability to utilize the tool while adhering to data sharing agreements, it might be beneficial to provide any further explicit assurances, e.g., guarantees that the limited access to data by core developers will strictly uphold privacy and not be utilized for any purpose beyond trouble shooting in cases where users request support. **We have added a privacy statement (as an additional tab in the main application) in which we elaborate on data privacy, and explicitly mention the data retention policy and the limited access to data as mentioned by the reviewer. We also stress that Z-scores are strictly speaking not anonymous and recommend that users pseudo-anonymize their data. We also detail how personal information on third-party software is handled (it is automatically and rapidly deleted).**

 The Figure 1 legend could briefly outline the purpose of the "test data" and "adaption data" fields to enhance user understanding and encourage immediate use. More detail regarding these fields and how to select appropriate data could be offered in the PCNportal’s "How to model" tab. **We have made this change by updating the Figure 1 legenda and updating PCNportal’s “How to Model” tab. In the tab, we explain how to best split the data and the trade-off in data split, but leave the choice of data set up to the user – as we think any data set that fits the model data template is appropriate.**

 The PCNportal “compute” tab could incorporate an optional interactive questionnaire to help guide algorithm selection based on the user's data and specific requirements. This could further streamline procedures and help clinical users justify the selection of a specific algorithm in their publications. **We thank the reviewer for pointing this out and will clarify the algorithm selection.  Although we believe such a questionnaire would be very useful, it may not be applicable to PCNportal yet because the algorithm selection is currently still trivial. We currently only support two algorithms, BLR and HBR, with HBR being slower and more accurate. We have now clarified this difference in ‘Model information’. However, as new algorithms come online, we will ensure to documentation the relative merits of different algorithms to guide the user of our software.**

 The brain atlas used for normative modeling and associated template csv files could be stated in the “How to model tab” to help users organise their regional surface outputs as suitable PCNportal inputs. **Thank you. We have now incorporated a brain atlas bullet point in the model-specific information in the dropdown menu of ‘Compute here’ upon choosing a model and refer to this in the How to Model section.**

 To further cater to the clinical user base, it is suggested that the outputs generated by the PCNportal be presented in csv instead of pickle files to facilitate seamless integration of the generated insights into other—non-python-based—workflows. Furthermore, each of the 7 outputs could be briefly explained in the “How to model” tab. **We have made this change so that .csv results are now provided instead of .pkl (in the future the user will be able to choose). We explain the outputs in ‘How to Model’ as requested.**

 To ensure proper acknowledgement, the “compute” tab could include specific citations that should be referenced in reports utilizing this tool. **Thank you. We have inserted a citation in the home tab as well as the ‘compute’ tab.**
